# Supplementary material for: Hydrogen sulfide protects against cisplatin-induced experimental nephrotoxicity in animal models: a systematic review and meta-analysis
Source: PeerJ. 2025 May 22;13:e19481. doi: 10.7717/peerj.19481 (PMC12103845; doi:10.7717/peerj.19481)
Supplement: Supplemental Information 1 [file peerj-13-19481-s001.docx]

Supplementary Figures


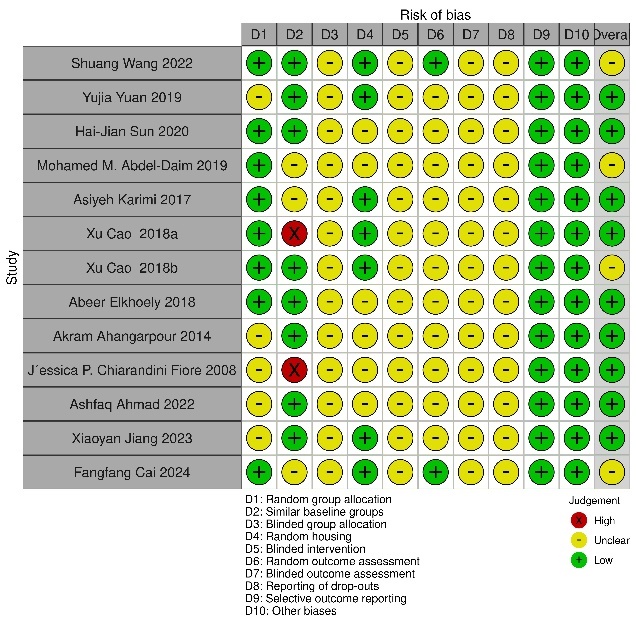
Supplementary Fig.1 Risk of Bias Assessment

Supplementary Fig.2 Sensitivity analysis.

In sensitivity analysis, the results remained consistent after excluding the studies one by one.


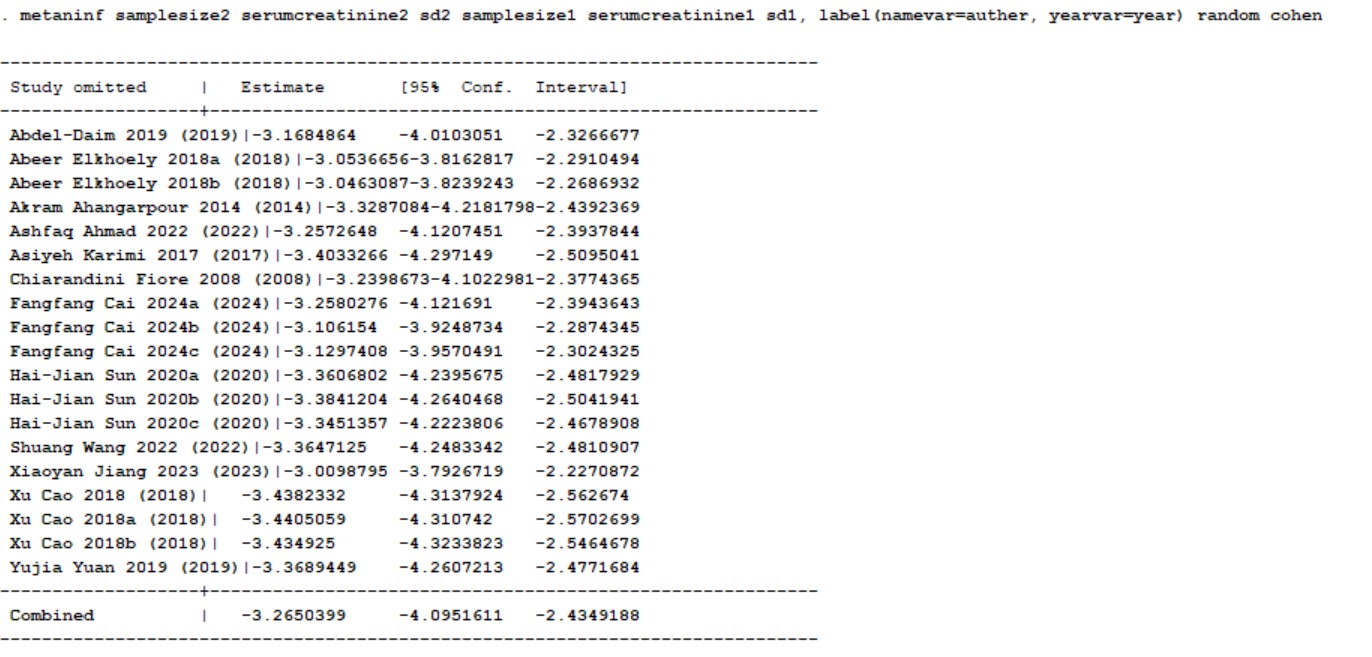


Supplementary Fig.3. Effects of hydrogen sulfide donor type on serum creatinine levels. Garlic Extract: Allicin, diallyl disulfide (DADS), diallyl sulfide (DAS), and diallyl trisulfide (DATS); ADT-OH: 5-(4-hydroxyphenyl)-3H-1,2-dithiocyclopentene-3-thione


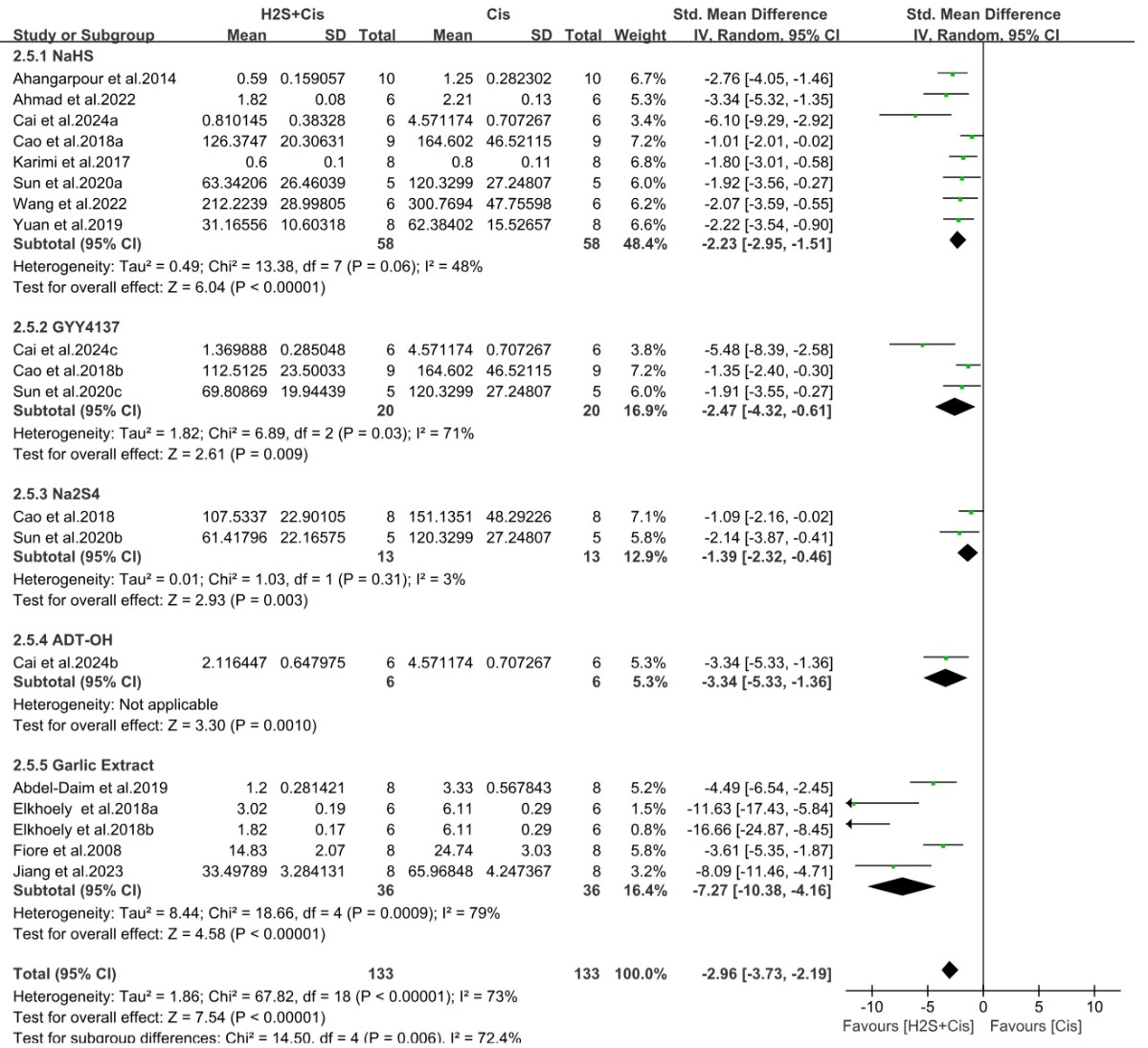


Supplementary Fig.4. Effects of animal species on serum creatinine levels.


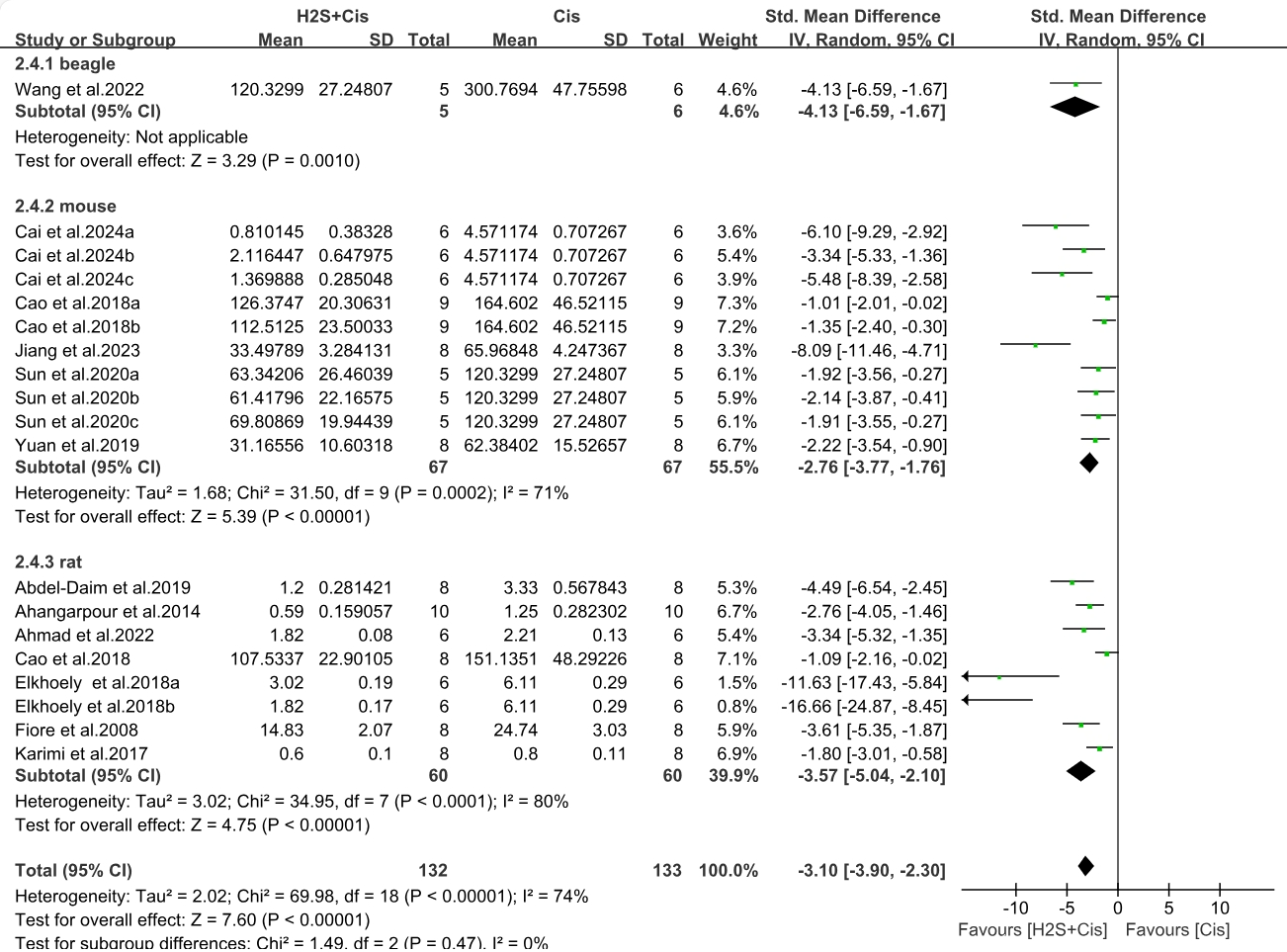


Supplementary Fig.5 Effects of administration method on serum creatinine levels. ip: intraperitoneal injection, ig: intragastric, iv: intravenous injection, po: peros(Latin)- oral administration.


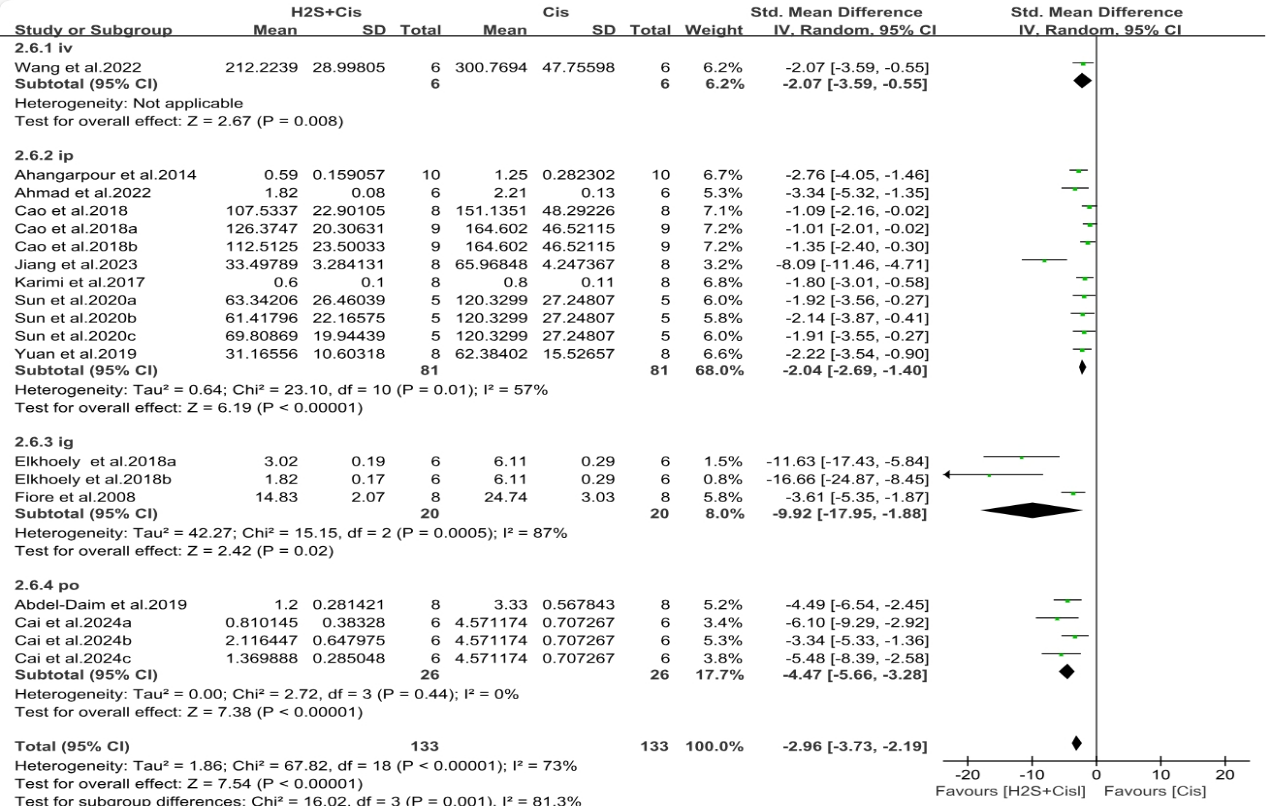


Supplementary Fig.6 The funnel plot represents the SMDs for serum creatinine level plotted against their standard error, with the vertical line representing the total summed estimates similar to the forest plot.


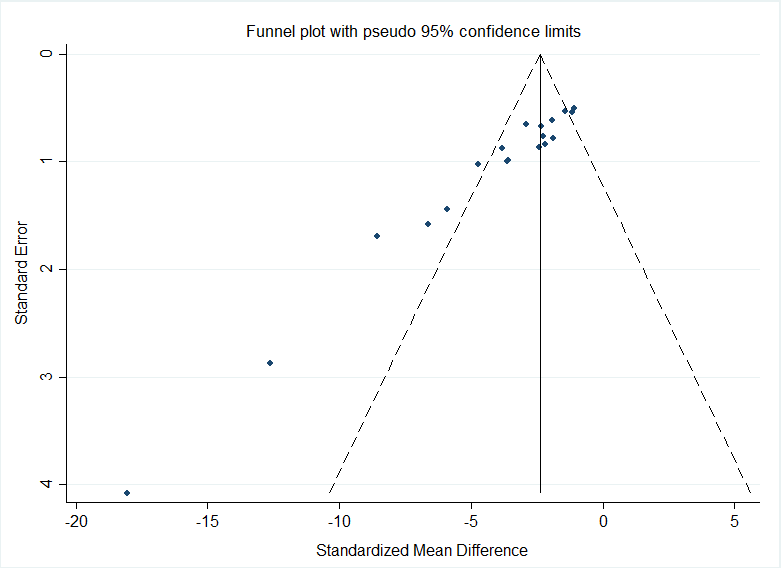


Supplementary Fig.7 Egger regression test


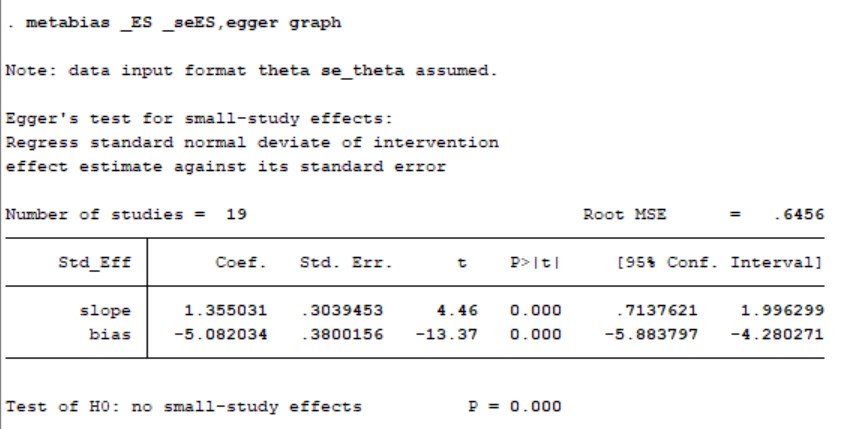


Supplementary Fig.8 After the application of the clipping method, the number of additions was 0.
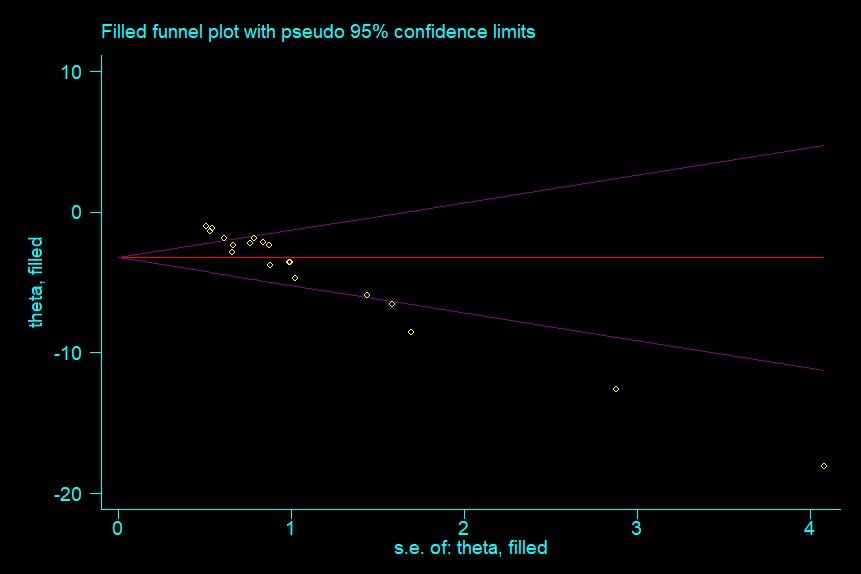


Supplementary Fig.9 Effects of hydrogen sulfide donor type on serum creatinine levels. 5


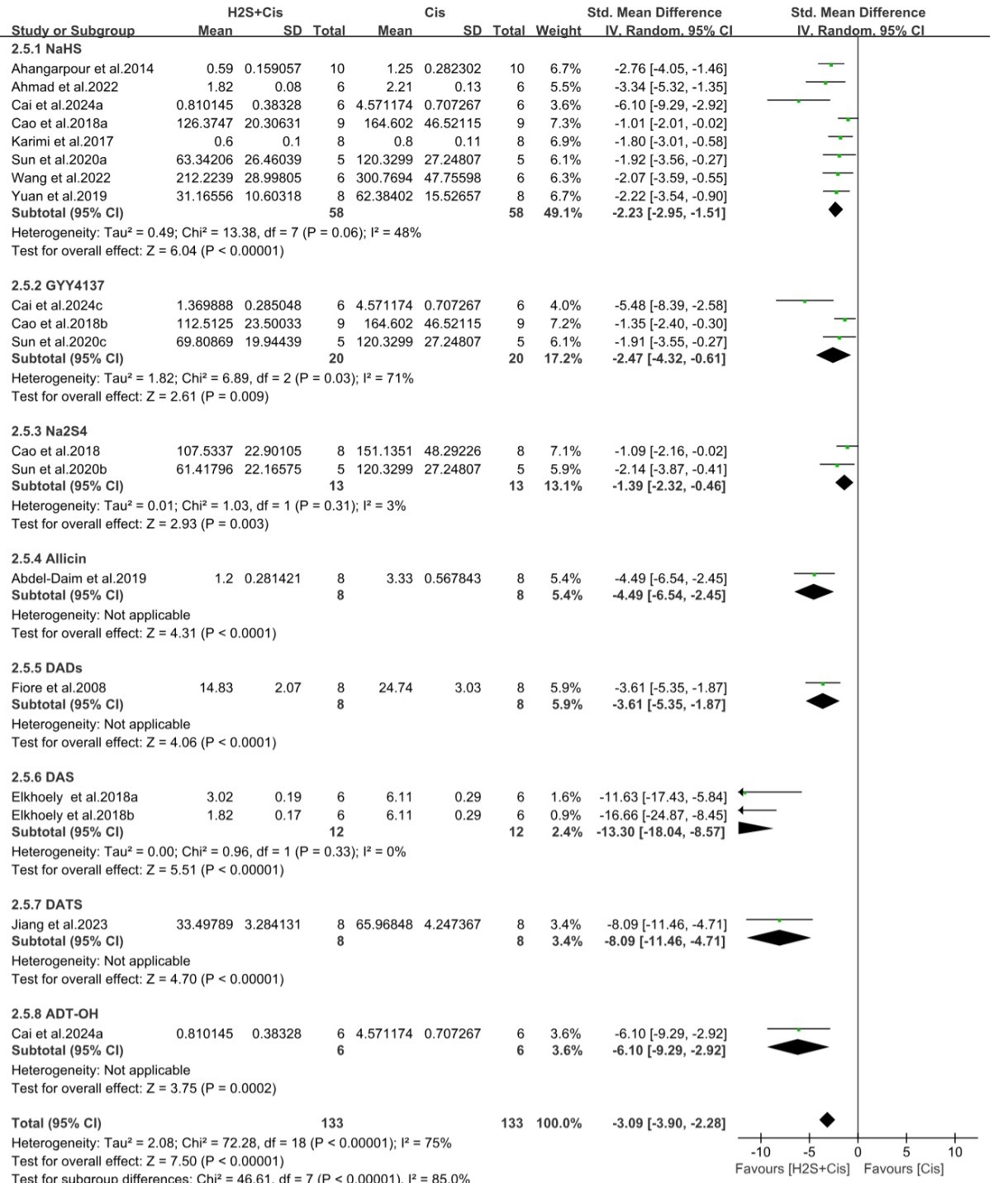


Search strategy

Keywords

1. Cisplatin

("Cisplatin"[Mesh]) OR ((((((((((((((cis-Diamminedichloroplatinum(II)) OR (Platinum Diamminodichloride)) OR (Diamminodichloride, Platinum)) OR (cis-Platinum)) OR (cis Platinum)) OR (Dichlorodiammineplatinum)) OR (cis-Diamminedichloroplatinum)) OR (cis Diamminedichloroplatinum)) OR (cis-Dichlorodiammineplatinum(II))) OR (NSC-119875)) OR (Platino)) OR (Platinol)) OR (Biocisplatinum)) OR (Platidiam))

2. Hydrogen Sulfide

("Hydrogen Sulfide"[Mesh]) OR ((((Sulfide, Hydrogen) OR (Hydrogen Sulfide (H2S3))) OR (Hydrogen Sulfide (H2(Sx)))) OR (Hydrogen Sulfide (H2S2)))

3. Acute Kidney Injury

("Acute Kidney Injury"[Mesh]) OR (((((((((((((((((((((((Acute Kidney Injuries) OR (Kidney Injuries, Acute)) OR (Kidney Injury, Acute)) OR (Acute Renal Injury)) OR (Acute Renal Injuries)) OR (Renal Injuries, Acute)) OR (Renal Injury, Acute)) OR (Renal Insufficiency, Acute)) OR (Acute Renal Insufficiencies)) OR (Renal Insufficiencies, Acute)) OR (Acute Renal Insufficiency)) OR (Kidney Insufficiency, Acute)) OR (Acute Kidney Insufficiencies)) OR (Kidney Insufficiencies, Acute)) OR (Acute Kidney Insufficiency)) OR (Kidney Failure, Acute)) OR (Acute Kidney Failures)) OR (Kidney Failures, Acute)) OR (Acute Renal Failure)) OR (Acute Renal Failures)) OR (Renal Failures, Acute)) OR (Renal Failure, Acute)) OR (Acute Kidney Failure))

Search Pubmed: **1 or 2**; 1 or 3; 1 or 2 or 3; 2 or 3 ; 1 and 2; 1 and 3; 1 and 2 and 3
